# Pancreatic Islet Cell Crosstalk: Insight Into α‐/β‐Cell Compensatory Mechanisms

**DOI:** 10.1002/cph4.70158

**Published:** 2026-05-01

**Authors:** Štěpánka Benáková, Blanka Holendová, Jurij Dolenšek, Monika Křivonosková, Andraž Stožer, Lydie Plecitá‐Hlavatá

**Affiliations:** ^1^ Department of Pancreatic Islet Research, Institute of Physiology Czech Academy of Sciences Prague Czech Republic; ^2^ Faculty of Medicine University of Maribor Maribor Slovenia

**Keywords:** cAMP signaling, diabetes, GCGR, GLP‐1R, insulin secretion, pancreatic islet, paracrine signaling

## Abstract

To investigate the compensatory role of α‐cell‐derived paracrine signaling through glucagon and GLP‐1 receptors in maintaining β‐cell function when insulin secretion is compromised. A β‐cell‐specific *Nox4* knockout mouse (*Nox4* ^βKO^) displays defective glucose‐stimulated insulin secretion and develops a prediabetic phenotype. To uncover the adaptive changes, we ran a detailed analysis of *Nox4* ^βKO^ pancreatic islets. We analyzed their composition, hormone secretion dynamics, receptor expression profiles, and downstream signaling pathways by immunocytochemistry, flow cytometry, RNA sequencing, cAMP assays, and insulin or glucagon secretion assays using both isolated islets and pancreatic slices across different glucose levels and receptor‐modulating conditions. Prediabetic *Nox4* ^βKO^ islets showed increased α‐cell numbers, expansion of bihormonal cells, and elevated production of glucagon and GLP‐1. Receptor profiling revealed a shift in receptor engagement: whereas GLP‐1R dominated in wild‐type islets, GCGR signaling gained prominence in *Nox4* ^βKO^ islets. This functional rebalancing is consistent with an adaptive response to emerging β‐cell dysfunction. Functional assays demonstrated that insulin secretion in prediabetic islets became increasingly reliant on glucagon‐driven potentiation of GLP‐1R and cAMP‐dependent pathways. Transcriptomic and signaling data confirmed enhanced expression of cAMP‐related intermediates and calcium‐handling components, indicating partial preservation of insulin secretory capacity despite underlying defects. α‐cell remodeling and flexible engagement of glucagon and GLP‐1 receptors act as key compensatory mechanisms that may help to sustain insulin secretion during early β‐cell stress. The context‐dependent plasticity of intra‐islet receptor activation highlights a coordinated multicellular adaptation in prediabetes and suggests that targeting intra‐islet endocrine crosstalk may help preserve β‐cell function in prediabetes.

## Graphical abstract

α‐cell remodeling compensates for impaired β‐cell function. Intra‐islet glucagon/GLP‐1 signaling is upregulated and becomes essential in prediabetic islets. Flexible receptor engagement reflects functional plasticity in hormonal control of insulin secretion. Created in BioRender. Plecita, L. (2026); https://BioRender.com/pgjjg3m.

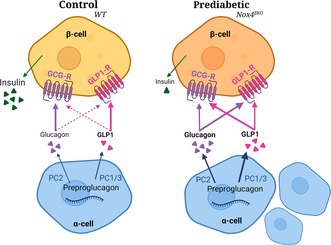

## 1 Introduction

Pancreatic islets maintain systemic nutrient homeostasis through coordinated interactions among β‐, α‐, and δ‐cells, mediated by paracrine signaling, electrical coupling, and adhesion contacts (Felix‐Martinez and Godinez‐Fernandez 2023; Hill and Hill 2024). These communication pathways enable precise regulation of hormone secretion. In healthy islets, β‐cells influence α‐cell activity by releasing signaling molecules such as insulin, GABA, serotonin, urocortin‐3, and zinc ions (Huising 2020). In turn, α‐cells contribute to islet homeostasis by secreting glucagon and the incretin hormone glucagon‐like peptide‐1 (GLP‐1), while δ‐cells release somatostatin to inhibit both insulin and glucagon. Electrical coupling via connexin 36 synchronizes β‐cell activity, supporting oscillatory electrical activity and calcium signaling essential for pulsatile insulin release (Serre‐Beinier et al. 2009). Disruption of these intercellular networks, particularly paracrine crosstalk, plays a central role in the pathogenesis of type 2 diabetes (T2D). Increasing evidence points to functional heterogeneity and plasticity within α‐ and β‐cell populations, which may underlie compensatory responses during metabolic stress (Bramswig et al. 2013).

α‐cells are no longer viewed as passive β‐cell antagonists. Instead, they actively contribute to islet regulation by secreting preproglucagon‐derived peptides, primarily glucagon and GLP‐1, which modulate insulin secretion. Their production is determined by prohormone convertase (PC) activity: PC2 favors glucagon generation, whereas PC1/3 promotes GLP‐1 production (Mojsov et al. 1986), particularly under metabolic stress (Mezza et al. 2025; Nie et al. 2000; Ellingsgaard et al. 2011). PC1/3 expression has been associated with a subpopulation of immature α‐cells (Saikia et al. 2021; O'Malley et al. 2014).

Unlike circulating GLP‐1, which is rapidly degraded by dipeptidyl peptidase IV (DPP‐IV), intra‐islet GLP‐1 may retain its paracrine activity due to the proximity between cells, allowing effective signaling despite local DPP‐IV presence (Evans and Wei 2022; Omar et al. 2014). GLP‐1 acts through its receptor (GLP‐1R) on β‐cells, triggering cAMP accumulation and activation of downstream pathways (PKA/EPAC/MEK–ERK/Wnt/β‐catenin) that enhance insulin exocytosis, survival, and proliferation (Dyachok et al. 2008; Campbell and Drucker 2013; Muller et al. 2019; Yusta et al. 2006). These cAMP‐induced effects often involve calcium signaling, enabling long‐term β‐cell adaptation under metabolic stress. Intriguingly, GLP‐1R activation can also enhance GLP‐1 expression in neighboring α‐cells (Saikia et al. 2021), and in mice, it can induce α‐cell transdifferentiation (Zhang et al. 2019). While this effect is most prominent in postprandial healthy islets, it is also proposed to function as a compensatory mechanism under metabolic stress. Supporting this, β‐cell‐specific GLP‐1R knockdown mice have normal oral glucose tolerance but impaired intraperitoneal glucose tolerance (Smith et al. 2014), indicating the physiological importance of intra‐islet GLP‐1R signaling.

Glucagon signals via its classical glucagon receptor (GCGR) on β‐cells to potentiate insulin secretion and, at high concentrations, can also partially activate GLP‐1R (Wei et al. 2023). Both GLP‐1 and glucagon thus support β‐cell function through overlapping, receptor‐dependent pathways, and effects amplified by receptor promiscuity (Shuai et al. 2021). Nonetheless, GLP‐1R appears to be the predominant contributor to cAMP‐mediated insulin secretion under physiological conditions, as its blockade markedly reduces the insulinotropic effect, even in the presence of elevated glucagon (Brown and Tzanakakis 2023).

This integrated signaling network demonstrates remarkable plasticity, allowing β‐cells to adjust insulin output in response to metabolic demands. In T2D, intracellular signaling within islets becomes dysregulated due to changes in hormone levels, receptor activity, and cell–cell communication (Zhang et al. 2021; Zeigerer et al. 2021). While compensatory adaptations are believed to occur in response to increased insulin demand, the molecular mechanisms that coordinate β‐α‐cell crosstalk under metabolic stress remain poorly understood.

To test how impaired β‐cell function influences α‐cell paracrine signaling, we used a mouse model of impaired GSIS based on β‐cell‐specific knockout of NADPH oxidase 4 (*Nox4* ^βKO^), an enzyme responsible for hydrogen peroxide production and redox signaling necessary for proper β‐cell physiological function and maintenance. We previously showed that *Nox4* ^βKO^ islets and animals exhibit blunted GSIS in vitro and in vivo following intraperitoneal glucose administration (Plecita‐Hlavata et al. 2020). These mice display a prediabetic phenotype, including insulin resistance and fat accumulation. Their limited ability to respond to nutrient stimuli, even during nutritional overload, leads to reduced food intake and prolonged intervals between feedings (Holendova, Benáková, et al. 2024). Despite these metabolic impairments, they maintain a normal lifespan, suggesting long‐term compensatory adaptations. Thus, the *Nox4* ^βKO^ model represents a physiologically relevant system to study intra‐islet compensatory mechanisms arising from β‐cell dysfunction.

## 2 Methods

### 2.1 Animals, Pancreatic Islet Isolation, and Experimental Procedure

Adult C57BL/6J (C57BL/6J; Ins2^*Cre−/−*^; Nox4^*flox/flox*^) and *Nox4* ^βKO^ mice (12–16 weeks) were housed at 22°C under a 12‐h light–dark cycle with free access to chow and water (Plecita‐Hlavata et al. 2020; Holendova, Benáková, et al. 2024). Procedures complied with EU directive 86/609/EEC and were approved by the Czech Central Commission for Animal Welfare.

Islets were isolated by collagenase IX digestion and ficoll gradient separation (Plecita‐Hlavata et al. 2020; Holendova, Benáková, et al. 2024; Holendova, Šalovská, et al. 2024) and islets from male and female mice were pooled equally for the analyses. For transcriptomics and cytometry, islets were cultured for 24 h in CMRL with 5.5 mM or 25 mM glucose; freshly isolated islets were used for cAMP assays.

### 2.2 Preparation of Pancreatic Slices and Insulin Secretion Analysis

Live 140 μm pancreatic slices were prepared as described previously (Stozer et al. 2013). Slices (five islets/well) were starved in KRH buffer (3 mM glucose) for 2 h; basal samples were collected, followed by a 1‐h incubation in KRH with 3 mM or 25 mM glucose ± Exendin‐4 (100 nM, MedChem, USA), Exendin (9–39) (100 nM, MedChem, USA), Crotedumab (2 nM, MedChem, USA), Glucagon (50 nM, MedChem, USA). Insulin and glucagon were analyzed by ELISA kits (Revvity, CrystalChem, USA).

### 2.3 Immunocytochemistry and Flow Cytometry

Endocrine cells dissociated with Acutase (Sigma‐Aldrich, USA) were fixed (BD Cytofix, BD Biosciences, USA), permeabilized (0.1% saponin), and stained with PE‐anti‐insulin (FAB1417P, Novus Biologicals, USA) and APC‐anti‐glucagon (FAB1249A, Novus Biologicals, USA) antibodies. Data were collected on an LSRII cytometer and analyzed using FlowJo v10.

### 2.4 Pancreatic Lysates for GLP‐1 Quantification

Pancreases were perfused with ice‐cold PBS containing sitagliptin (200 nM, MedChem, USA), excised, snap‐frozen, and homogenized. GLP‐1 content was measured using ELISA (EZGLPHS, Millipore, USA); BSA/PBS served as matrix control.

### 2.5 Immunochemical Semi‐Quantification

Equal protein amounts were separated by SDS‐PAGE, transferred to PVDF, and probed for insulin (ab181547, Abcam, UK), GCGR (ab75240, Abcam, UK), and GLP‐1R (BS‐1559R, Bioss, ThermoFisher Scientific, USA). Bands were quantified by ImageJ and normalized to total protein (Schneider et al. 2012).

### 2.6 Histology and Immunofluorescence Staining

Tissue samples were formalin‐fixed, paraffin‐embedded, and sectioned at 4 μm. Sections were stained with H&E or immunolabeled for insulin and glucagon using anti‐insulin (ab181547, Abcam) and anti‐glucagon (14‐9743‐82, Thermo Fisher Scientific) antibodies and mounted in DAPI‐containing medium.

### 2.7 Islet Size Analysis

Paraffin‐embedded pancreas sections (40 μm) collected at regular intervals were stained with H&E. Images were acquired on a Leica SP8 microscope, and islet cross‐sectional area was quantified using ImageJ (Schneider et al. 2012).

### 2.8 RNA Extraction, cDNA Synthesis, and RT‐qPCR

RNA from islets or INS1 cells was isolated using the RNeasy kit (Qiagen, Germany) and reverse‐transcribed (TATAA Biocenter, Sweden). qPCR was performed using EvaGreen dye (Biotium, USA), and relative expression was calculated by the 2^−ΔΔ*Ct*^ method using a combination of *Rplp0*, *Ppia*, *Hprt*, and *Ywhaz* (mouse) or *Ywhaz* (INS1) as reference genes validated by NormFinder/geNorm (Figure S1B,C).

### 2.9 RNA Sequencing

RNA sequencing was conducted as previously outlined (Holendova, Šalovská, et al. 2024). The statistical significance of the RNA‐seq data was determined using the edgeR package, with a negative binomial model and Benjamini‐Hochberg correction for multiple testing. The values shown in the figures as “read counts” are normalized values that are consistent with the model applied for differential expression analysis. The RNAseq dataset has been submitted to the NCBI GEO repository under the GEO Series GSE274980 (Holendova, Šalovská, et al. 2024), GSE319574 (secure token to view series while it remains in private status: gnyzekcybbidfwt) and GSE307298 (secure token to view series while it remains in private status: glmpwsiwfjehlsp). Expression changes of selected genes were validated by quantitative RT‐PCR (Table S1, Figure S1).

### 2.10 cAMP Detection

cAMP levels were measured using an HTRF detection kit (62AM6PEB, Revvity, USA), with forskolin (MedChem, USA) as a positive control.

### 2.11 Oral Glucose Tolerance Test and Insulin Secretion

After an overnight fast, mice received 2 g/kg an oral glucose. Blood glucose and serum insulin were measured at the indicated time points (10, 15, 20, 60, and 120 min).

### 2.12 Calcium Imaging Analysis

Pancreas tissue slices were incubated with Calbryte 520 AM dye (AAT Bioquest) and imaged using confocal microscopy (Leica TCS SP5 AOBS, Leica SP8 Stellaris) while perfused with a carbogenated ECS physiological solution at 37°C–40°C. Glucose stimulation involved basal (6 mM) and elevated (8, 12, 16 mM) concentrations. Calcium signals from individual β‐cells were recorded, binarized for oscillation events, and analyzed to measure active time and coactivity (synchrony) during the transient 1st and sustained 2nd phase of response. Analysis followed established protocols from previous studies (Dolensek et al. 2013; Pohorec et al. 2022; Stozer et al. 2013, 2021), with more details in Supporting Information.

### 2.13 Statistical Analysis

RNAseq included three biological replicates per group (islets pooled from four mice to create one replicate). Other experiments used three to eight biological replicates per group when isolated islets were used (islets pooled from three mice to create one replicate) or in the case of using pancreatic slices, four to seven biological replicates were used (one replicate counted 5 islets within slices). For the histological analysis of islet size, pancreatic sections obtained from three mice per group were analyzed. For analyses of impaired insulin secretion and glucose tolerance in mice, six to ten biological replicates were used, each derived from an individual mouse. Data were analyzed using ANOVA with Tukey/Sidak tests or Student's *t*‐test; calcium imaging used Mann–Whitney tests. *p* < 0.05 was considered significant.

The AIGC tool (ChatGPT) was used to edit and correct the language.

## 3 Results

### 3.1 Impaired Insulin Secretion and Calcium Dynamics in *Nox4* ^βKO^ Islets

**FIGURE 1:**
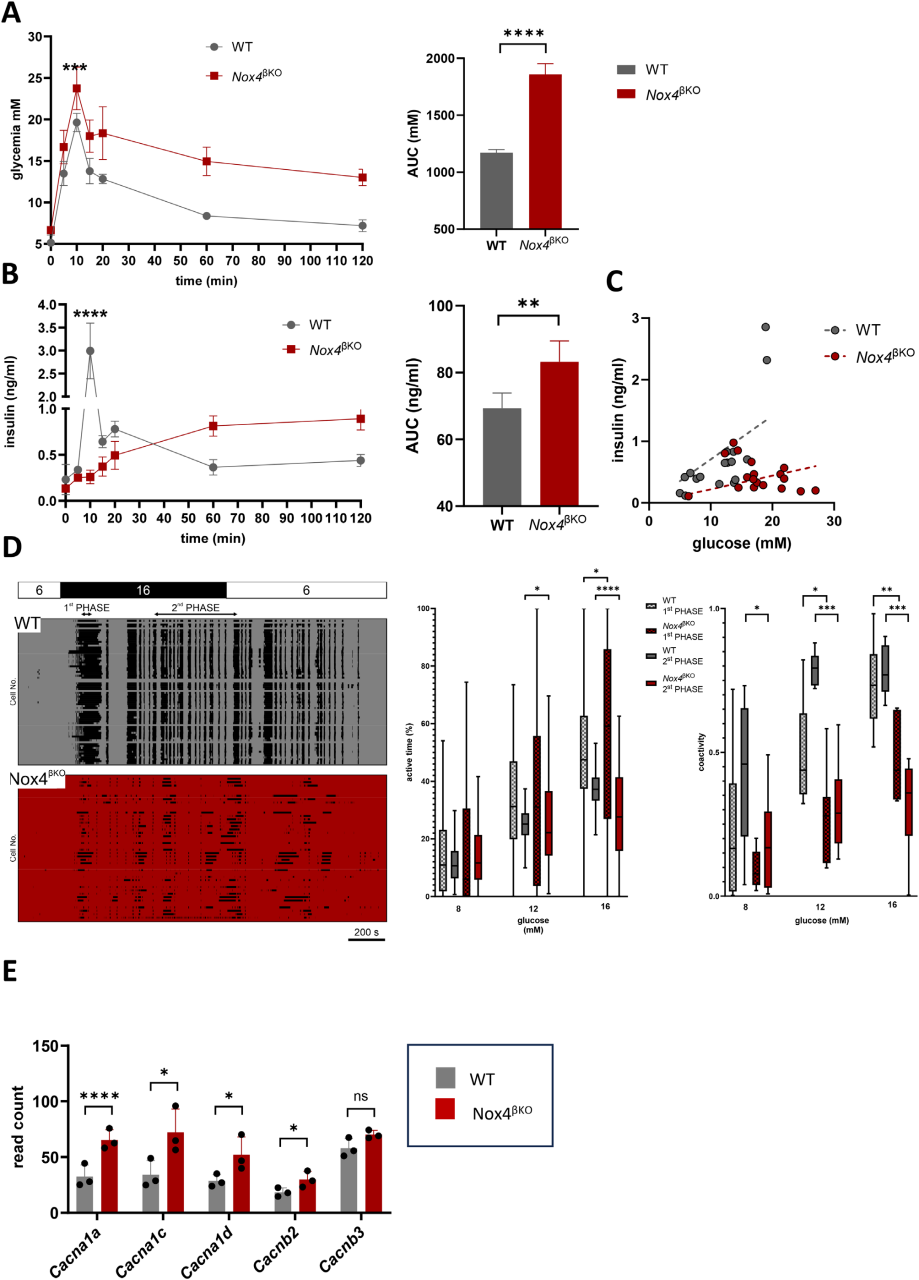
Characterization of insulin secretion of prediabetic model. (A) Oral glucose tolerance test‐ glucose quantification (A) and insulin quantification (B) of WT (gray circles) and *Nox4* ^βKO^ animals (red squares). Next to time‐point course are calculated AUC; *n* = 6–10, glycemia (ANOVA, *p* = 0.003, the rest n.s.); insulin (ANOVA, *p* < 0.0001, the rest n.s.), AUC glycemia (ANOVA, *p* < 0.0001), AUC insulin (ANOVA, *p* = 0.0012). (C) Paired values from glucose (panel A) and insulin (panel B) were plotted for WT (gray) and *Nox4* ^βKO^ (red); *n* = 6–10. Linear regression is indicated with broken lines. (D) β‐cell binarized activity for a typical islet from WT (left panel, gray rectangle) and *Nox4* ^βKO^ (left panel, red rectangle), binarization was based on calcium dynamics. Stimulatory protocol and dedicated intervals for analysis of the 1st and plateau phases are indicated above. We quantified percent active time (middle panel) and coactivity (right panel) during stimulation with 8, 12 or 16 mM glucose. Data is shown for the 1st (hatched boxplots) and the 2nd (solid boxplots) phases of glucose response (WT in gray and Nox4^βKO^ in red). *n* = 6, Mann–Whitney test, *p* = 0.03, *p* = 0.01, *p* < 0.0001 (middle panel) and *p* = 0.03, *p* = 0.01, *p* = 0.0002, *p* = 0.008, *p* = 0.0002 (right panel). (E) Quantification of calcium channels transcripts of WT (gray) and *Nox4* ^βKO^ islets (red); *n* = 3, ANOVA, *p* < 0.0001, *p* < 0.0001, *p* < 0.0001, *p* = 0.0135, *p* < 0.0001. *p ≤ 0.05, **p ≤ 0.01, ***p ≤ 0.001, ****p ≤ 0.0001.

To study intra‐islet endocrine communication during impaired insulin secretion, we employed β‐cell‐specific *Nox4* knockout mice (*Nox4* ^βKO^). To mimic physiological nutrient absorption, we performed an oral glucose tolerance test with insulin secretion analysis (Figure 1A,B). *Nox4* ^βKO^ mice showed impaired insulin release, particularly during the first phase of GSIS, consistent with reduced β‐cell responsiveness. Plasma insulin concentration expressed per glucose concentration further corroborated impaired insulin release in *Nox4* ^βKO^ (Figure 1C).

To gain insight into the functional changes in these animals, we analyzed β‐cell calcium dynamics (Figure 1D). Increasing glucose concentrations increased β‐cell active time during both the 1st and 2nd phases. However, the 2nd phase active time was reduced in *Nox4* ^βKO^ animals at higher glucose concentrations, further corroborating in vivo data (Figure 1C). Moreover, β‐cell coactivity (a functional measure of intercellular synchronization) demonstrated a clear glucose dependence in both WT and *Nox4* ^βKO^ mice, but was decreased in *Nox4* ^βKO^ β‐cells during both the 1st and 2nd phases (Figure 1D). We next examined the expression of voltage‐gated calcium channel subunits in these animals. In contrast to the functional defect, transcript levels of multiple L‐type and P/Q‐type subunits (*Cacna1a*, *Cacna1c*, *Cacna1d*, and *Cacnab2*) were strongly upregulated in *Nox4* ^βKO^ mouse islets (Figure 1E). The marked reduction in insulin secretion, particularly during the first phase, despite a comparatively smaller reduction in active time, is consistent with the secretory defect not being fully explained by altered calcium dynamics alone. Notably, the concurrent upregulation of voltage‐gated calcium channel transcripts may reflect transcriptional compensation downstream of enhanced intra‐islet GLP‐1R/cAMP signaling, as discussed further below.

### 3.2 Altered Islet Cell Composition and Presence of Bihormonal Cells in *Nox4* ^βKO^ Mice

**FIGURE 2:**
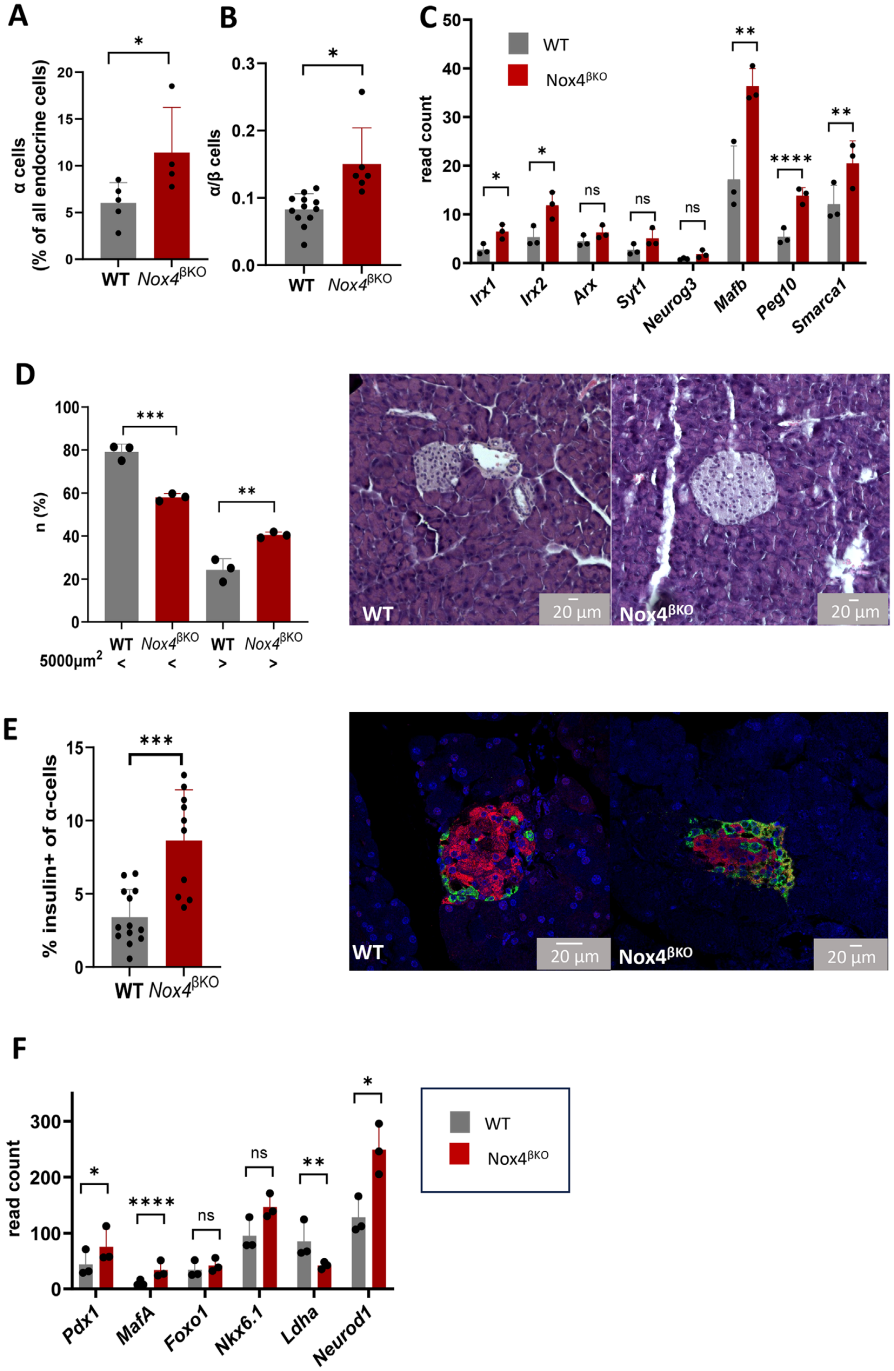
Quantification of α‐cells and bihormonality in prediabetic islets. (A) Number of α‐cells (glucagon positive cells) in all endocrine cells (*n* = 4–5) and (B) as α/β ratio (*n* = 6–12) in WT (gray) and *Nox4* ^βKO^ islets (red), *T*‐test, *p* = 0.0317, *p* = 0.0127. (C) Quantification of α‐specific transcripts in WT (gray) and *Nox4* ^βKO^ (red) islets (*n* = 3), statistical analysis was performed using a model for differential gene expression suited to RNA‐seq data (edgeR package), *p* = 0.0327, *p* = 0.0378, *p* = 0.202, *p* = 0.0553, *p* = 0.0587, *p* = 0.00517, *p* < 0.0001, *p* = 0.00961. (D) Quantification of small (< 5000μm^2^) and large (> 5000μm^2^) islets of WT (gray) and *Nox4* ^βKO^ (red) mice (*n* = 3), ANOVA, *p* = 0.0003, *p* = 0.0016. Representative figures are presented. (E) Quantification of bihormonal α‐cells (% of insulin positive α‐cells) (left panel) (*n* = 10–13), *T*‐test, *p* = 0.0008. Representative figures of the presence of bihormonal cells were performed by immunohistochemistry; and (F) quantification of β‐cell specific transcripts (identity transcripts‐*Pdx1*, *Foxo1*, *Nkx6.1* and disallowed transcript‐*Ldha*, and common maturity regulator in the pancreatic islet *Neurod1*) in WT (gray) and *Nox4* ^βKO^ (red) islets (*n* = 3). Statistical analysis was conducted using edgeR, a model designed for RNA‐seq data, *p* = 0.0481, *p* < 0.0001, *p* = 0.558, *p* = 0.236, *p* = 0.00264, *p* = 0.027. *p ≤ 0.05, **p ≤ 0.01, ***p ≤ 0.001, ****p ≤ 0.0001.

To further characterize changes in islet cell populations, we examined presence of α‐cells in *Nox4* ^*βKO*^ islets by flow cytometry. We found an increase in their numbers among all endocrine cells (Figure 2A), especially relative to the β‐cells (Figure 2B). This corresponds with increased expression of several α‐cell‐specific transcripts, including significantly upregulated genes such as *Irx1*, *Irx2*, *Mafb*, *Peg10* and *Smarca1*, while others (e.g., *Arx Syt1*, *Neurog3*) showed no significant change as revealed by islets RNAseq analysis (Figure 2C). Interestingly, immunohistochemical analysis of pancreatic islets showed an increased number of larger islets (over 5000 μm^2^, corresponding to approximately 40 μm in diameter), while the number of smaller islets (below 5000 μm^2^) was decreased (Figure 2D). Further analysis showed an increased number of bihormonal (insulin‐ and glucagon‐positive) cells in *Nox4* ^βKO^ islets (Figure 2E). Despite increased α‐cell numbers, β‐cell‐specific transcripts (*Pdx1*, *MafA*) were increased, as well as *NeuroD1*, a pan‐endocrine transcription factor involved in the development of multiple islet cell types, while the dedifferentiation marker *Ldha* was reduced in *Nox4* ^*βKO*^ islets (Figure 2F). These findings indicate that β‐cell marker expression is largely preserved despite impaired insulin secretion, while the proportion of glucagon‐positive cells increases, including a subset of insulin/glucagon double‐positive endocrine cells.

### 3.3 Upregulation of Glucagon‐Related Signaling in Prediabetic *Nox4* ^βKO^ Islets

**FIGURE 3:**
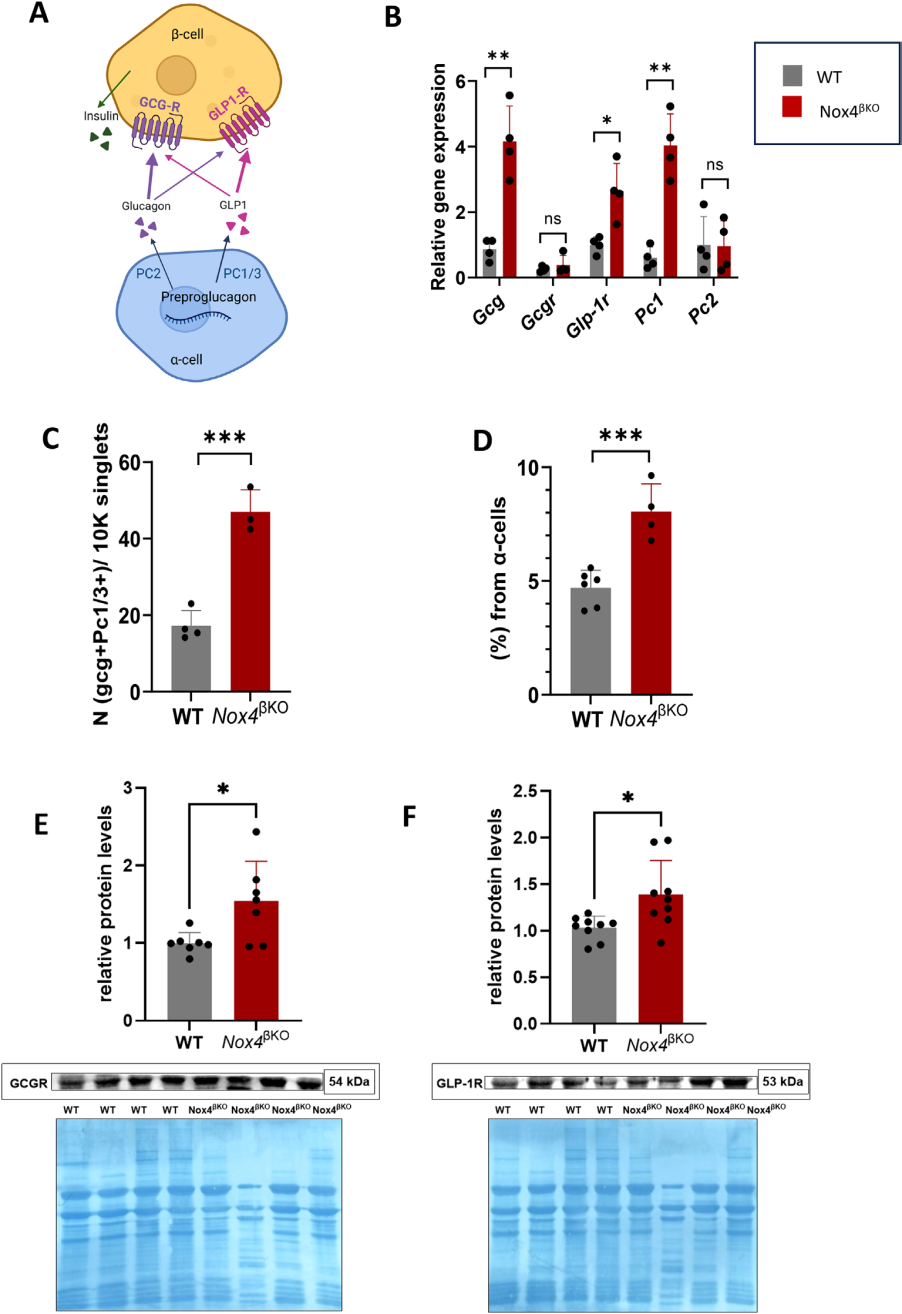
Quantification of glucagon/GLP‐1 linked transcripts/proteins. (A) Schema of paracrine communication of α‐/β‐cells through glucagon and GLP‐1. Created in Biorender. Plecita, L. (2026), https://BioRender.com/0mxd7q7. (B) qPCR quantification of preproglucagon‐related transcripts in WT (gray) and *Nox4* ^βKO^ (red) islets (*n* = 4), *T*‐test (Welch's correction), *p* = 0.0062, *p* = 0.4661, *p* = 0.0257, *p* = 0.0034, *p* = 0.9534. (C) Quantification of PC1/3 positive cells in WT (gray) and *Nox4* ^βKO^ (red) islets (*n* = 3–4), *T*‐test, *p* = 0.0007; and (D) of PC1/3 positive α‐cells of WT (gray) and *Nox4* ^βKO^ (red) islets (right panel) (*n* = 4–6), *T*‐test, *p* = 0.0005. (E) Quantification of GCGR relative protein levels in WT (gray) and *Nox4* ^βKO^ (red) pancreatic lysates (*n* = 7), *T*‐test, *p* = 0.0202. Representative blots are presented (F) Quantification of GLP‐1R relative protein in WT (gray) and *Nox4* ^βKO^ (red) pancreatic lysates (*n* = 9), *T*‐test, *p* = 0.0130. *p ≤ 0.05, **p ≤ 0.01, ***p ≤ 0.001, ****p ≤ 0.0001.

To assess whether paracrine signaling from α‐cells is altered in the setting of β‐dysfunction, we examined the expression of preproglucagon products and their receptors in prediabetic *Nox4* ^βKO^ islets. Preproglucagon in α‐cells can be processed into glucagon by PC2 and/or into GLP‐1 by PC1/3, which act on β‐cells through GCGR and GLP‐1R, respectively. However, these receptors were suggested to display some promiscuity for their agonists (Figure 3A).

We found that *Gcg* transcript levels are significantly increased in prediabetic *Nox4* ^*βKO*^ islets (3.5‐fold; Figure 3B, qPCR validation of RNAseq results in Figure S1A), whereas α‐cell numbers increased only 1.8‐fold (Figure 2A,B), indicating disproportionate *Gcg* upregulation relative to α‐cell expansion. This was accompanied by increased expression of *Pc1* (*Pcsk1*), suggesting enhanced proglucagon processing capacity in *Nox4* ^βKO^ islets. In contrast, the expression of *Pc2* (*Pcsk2*) and the glucagon receptor (*Gcgr*) remained unchanged. Interestingly, we observed a marked upregulation of the *Glp1r* transcript, which may reflect altered incretin signaling in the prediabetic state. Immunostaining further revealed an increase in the presence of glucagon and PC1/3‐double‐positive cells (Figure 3C), though the proportion of PC1/3‐positive α‐cells remained relatively low (8% vs. 4.7% in control islets; Figure 3D).

At the protein level, a modest yet statistically significant increase in GCGR expression was detected (Figure 3E), paralleled by a similar upregulation of GLP‐1R (Figure 3F). Densitometric quantification was normalized to total protein load, assessed by Coomassie Brilliant Blue staining, to ensure accurate comparison across samples. To assess whether the observed receptor upregulation could be attributed directly to the altered redox environment characteristic of *Nox4* ^βKO^ β‐cells, we examined *Gcg*, *Gcgr*, and *Glp1r* transcript levels in INS‐1 cells exposed to mild pro‐oxidative conditions—either glucose oxidase (GOX) or menadione—at non‐stimulatory glucose. Neither treatment altered *Gcgr* or *Glp1r* mRNA levels, while *Gcg* transcript was increased by stimulatory glucose but not by oxidative stress per se (Figure S2A–C). Interestingly, *Adcy3*, an adenylyl cyclase isoform was found downregulated in *Nox4* ^βKO^ islets, showed redox‐sensitive suppression under menadione treatment, whereas the effect of GOX was not significant (Figure S2D), suggesting that an altered redox environment may contribute to the decrease in Adcy3 expression in *Nox4* ^βKO^ β‐cells. Together, these data indicate that the upregulation of glucagon‐related receptors in prediabetic islets is unlikely to reflect a direct transcriptional response to oxidative stress, and may instead arise from broader islet remodeling associated with chronic β‐cell dysfunction. Proteomic analysis further showed no significant difference in cysteine oxidation status of these receptors between genotypes (data not shown) (Holendova, Šalovská, et al. 2024).

### 3.4 Insulin Secretion in Prediabetic Islets Depends on GLP‐1 Signaling via GCGR and Glucagon Signaling via GLP‐1R

**FIGURE 4:**
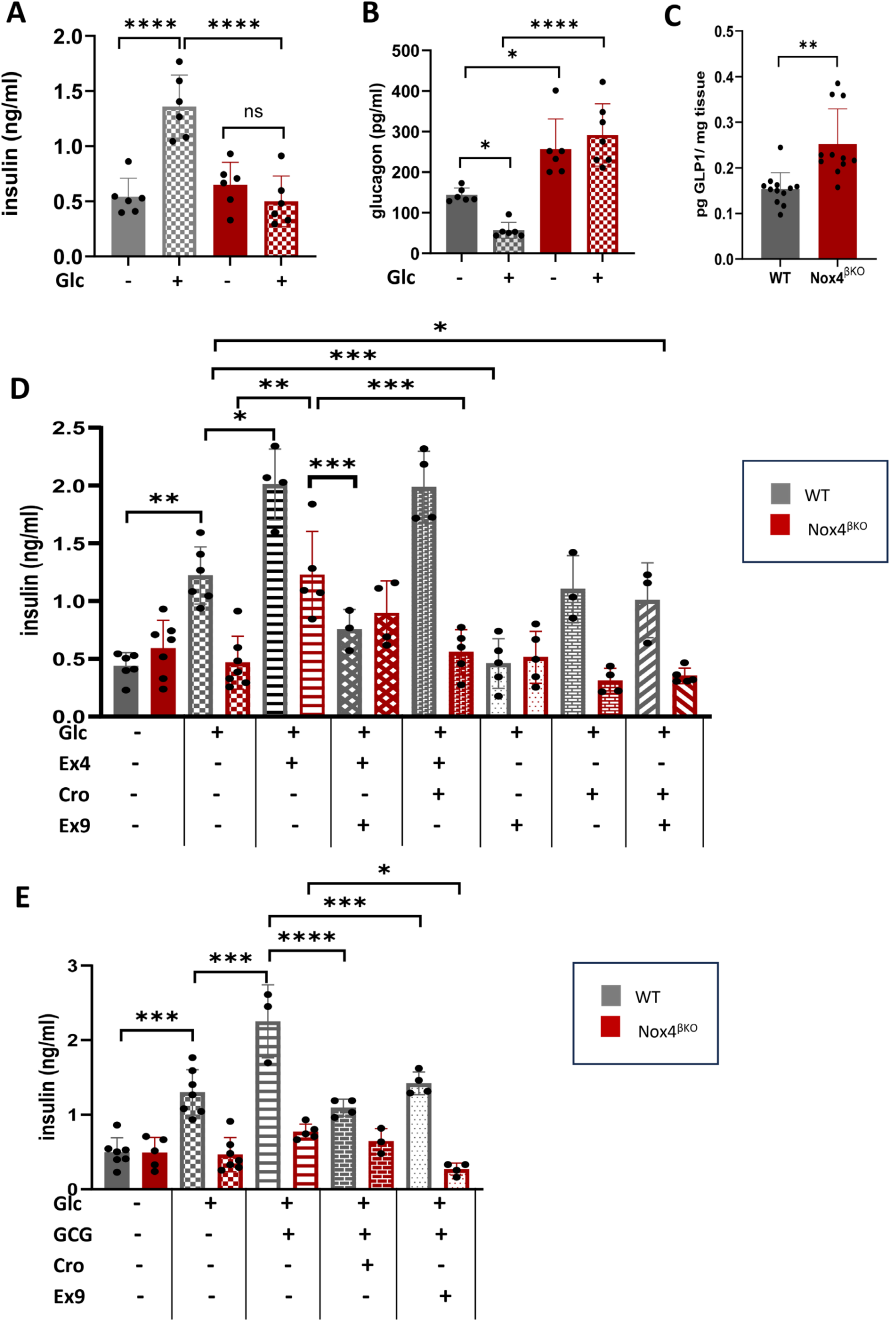
Functional analysis of prediabetic islets while manipulating GCG/GLP‐1 signaling. (A) Quantification of insulin secretion of WT (gray) and *Nox4* ^βKO^ (red) islets upon non‐stimulating (solid colors) or glucose‐stimulating (pattern colors) conditions (*n* = 6), ANOVA, *p* < 0.0001, *p* < 0.0001, *p* = 0.8421. (B) Quantification of glucagon secretion of WT (gray) and *Nox4* ^βKO^ (red) islets upon non‐stimulating (solid colors) or glucose‐stimulating (pattern colors) conditions (*n* = 6–7), ANOVA, *p* = 0.0112, *p* = 0.0523, *p* < 0.0001. (C) Quantification of GLP‐1 of WT (gray) and *Nox4* ^βKO^ (red) pancreases (*n* = 11–12), *T*‐test, *p* = 0.0018. (D) Analysis of insulin secretion of WT islets (gray labeling) and *Nox4* ^βKO^ islets (red labeling) upon non‐stimulating condition (solid color), stimulation by glucose (crosshatch); glucose and GLP‐1 (stripes); glucose, GLP‐1 and GLP‐1R antagonist (Ex9) (diagonal grid); glucose, GLP‐1 and GCGR antagonist (Cro) (filled bricks); glucose, and GLP‐1R antagonist (Ex9) (light dots); glucose, and GCGR antagonist (Cro) (light bricks) and glucose; GLP‐1R antagonist (Ex9), and GCGR antagonist (Cro) (diagonal stripes), (*n* = 3–7), ANOVA, for WT islets: *p* = 0.008, *p* = 0.0140, *p* = 0.0008, *p* = 0.7268, *p* = 0.0006, *p* = 0.9143, *p* = 0.0255; for *Nox4* ^βKO^ islets: *p* = 0.9723, *p* = 0.0013, *p* = 0.4586, *p* = 0.0008, *p* = 0.4112, *p* = 0.2657, *p* = 0.7645. Only significant statistics for the relevant samples are presented. (E) Analysis of insulin secretion of WT islets (gray labeling) and *Nox4* ^βKO^ islets (red labeling) upon non‐stimulating condition (solid color), stimulation by glucose (crosshatch); glucose and glucagon (stripes); glucose, glucagon and GCGR antagonist (Cro) (bricks); glucose, glucagon and GLP‐1R antagonist (Ex9) (light dots); (*n* = 3–7), ANOVA, for WT islets: *p* = 0.0001, *p* = 0.0001, *p* < 0.0001, *p* = 0.0003 and for *Nox4* ^βKO^ islets: *p* = 0.9990, *p* = 0.7679, *p* = 0.3741, *p* = 0.0315. Only significant statistics for the relevant samples are presented. *p ≤ 0.05, **p ≤ 0.01, ***p ≤ 0.001, ****p ≤ 0.0001.

To assess the functional impact of upregulated glucagon/GLP‐1 signaling, we measured insulin and glucagon secretion in pancreatic slices under both stimulatory (16 mM glucose) and non‐stimulatory (3 mM glucose) conditions, and quantified total GLP‐1 levels. In WT islets, glucose increased insulin and simultaneously suppressed glucagon release, whereas *Nox4* ^βKO^ islets displayed suppressed insulin and paradoxically increased glucagon secretion under both conditions (Figure 4A,B). Notably, glucagon release was already elevated under basal (non‐stimulatory) conditions and remained high upon glucose stimulation (Figure 4B). To evaluate intra‐islet GLP‐1 production, we quantified total GLP‐1 protein from whole pancreatic lysates. Despite the typically low abundance and instability of GLP‐1 protein, levels were significantly elevated in *Nox4* ^βKO^ mice, suggesting enhanced α‐cell GLP‐1 output (Figure 4C).

To clarify the contributions of GLP‐1R and GCGR signaling to GSIS, we treated islets in pancreatic slices with the GLP‐1 mimetic exendin‐4, the GLP‐1R antagonist exendin‐9‐39 (both of which bind to the same site on GLP‐1R), and the GCGR‐blocker crotedumab (IgG4 monoclonal antibody). Insulin secretion was assessed under glucose stimulation with or without these modulators in vitro (Figure 4D). In WT islets, insulin secretion was enhanced by exendin‐4 and reduced by exendin‐9‐39, while GCGR inhibition had minimal effect, confirming GLP‐1R dependence. In contrast, *Nox4* ^βKO^ islets responded more strongly to exendin‐4, and this effect was more effectively attenuated by GCGR blockade than GLP‐1R inhibition, consistent with altered receptor engagement.

Glucagon application increased GSIS in WT islets, and this effect was attenuated by both exendin‐9‐39 and crotedumab (Figure 4E). In *Nox4* ^βKO^ islets, however, glucagon failed to enhance GSIS. Yet, GLP‐1R inhibition markedly reduced insulin secretion, while GCGR blockade had no effect (Figure 4E). These patterns were also observed with glucose stimulation and inhibitors alone, though to a lesser extent than with the GLP1 mimetic or glucagon stimulation due to low sensitivity of the current commercial ELISA.

To further investigate receptor preference on β‐cells, MIN6 and INS‐1 clonal insulin‐secreting lines were treated with glucagon under stimulatory glucose conditions. In both lines, GLP‐1R inhibition suppressed insulin secretion, whereas GCGR inhibition did not (Figure S3).

Together, these results are consistent with a context‐dependent shift in receptor engagement: in WT islets, GLP‐1R signaling appeared to predominate, whereas in *Nox4* ^βKO^ islets the functional data suggest a pattern of altered receptor utilization in which GLP‐1‐associated effects may be partially mediated via GCGR, and glucagon‐associated effects via GLP‐1R. Whether this reflects true receptor cross‐activation, altered ligand availability, or biased downstream signaling remains to be determined. cAMP signaling downstream of GLP‐1R and GCGR is reprogrammed in prediabetic islets.

The GLP‐1R and GCGR both primarily activate cyclic AMP (cAMP) signaling cascades, which regulate insulin secretion, β‐cell function, and survival. To evaluate cAMP signaling dynamics in islets, we measured intracellular cAMP levels under glucose stimulation and in response to receptor‐specific modulators.

**FIGURE 5:**
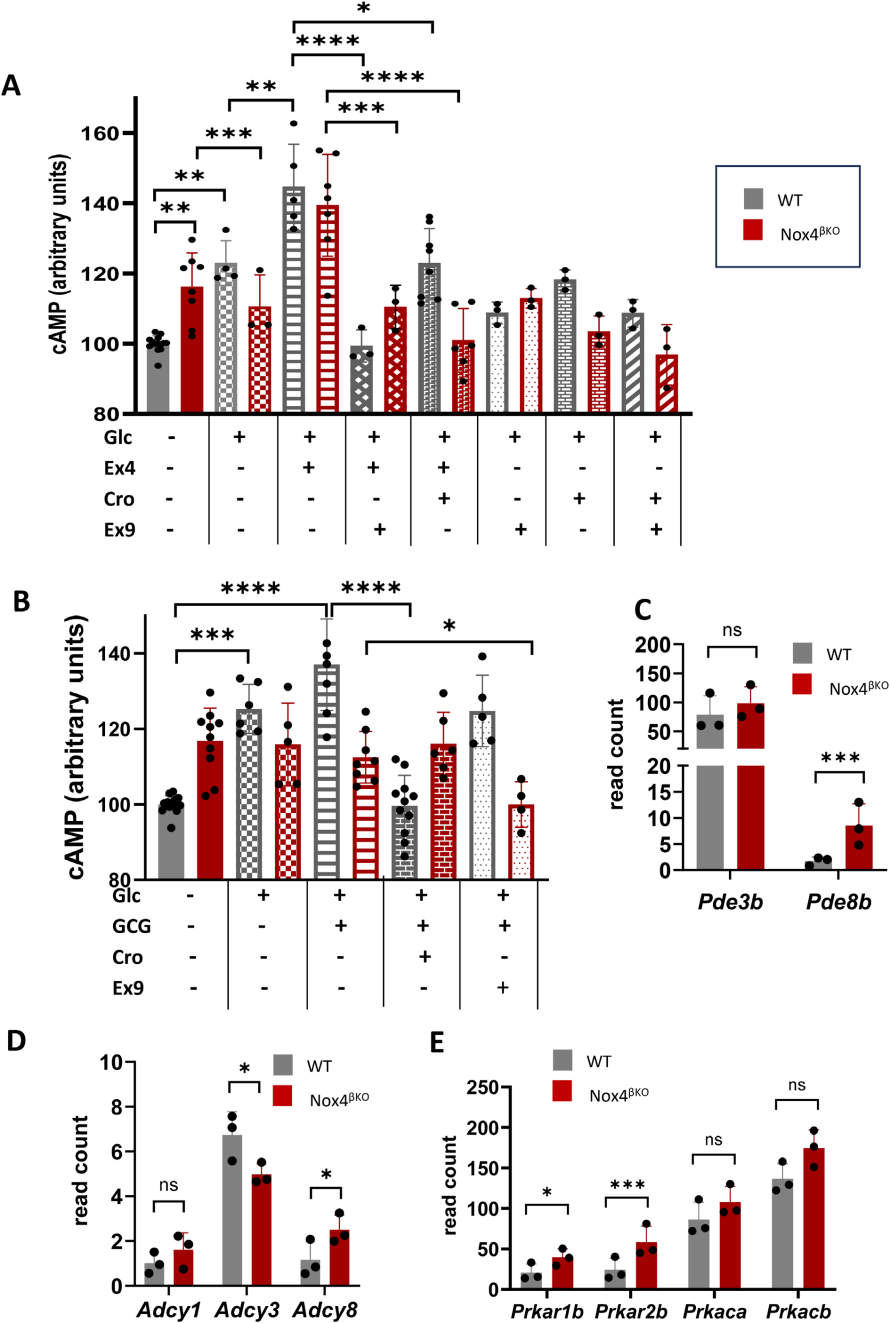
Quantification of cAMP signaling in prediabetic islets. (A) cAMP quantification in WT islets (gray labeling) and *Nox4* ^βKO^ islets (red labeling) upon non‐stimulating condition (solid color), stimulation by glucose (crosshatch); glucose and GLP‐1 (stripes); glucose, GLP‐1 and GLP‐1R antagonist (Ex9) (diagonal grid); glucose, GLP‐1 and GCGR antagonist (Cro) (filled bricks); glucose, and GLP‐1R antagonist (Ex9) (light dots); glucose, and GCGR antagonist (Cro) (light bricks) and glucose; GLP‐1R antagonist (Ex9), and GCGR antagonist (Cro) (diagonal stripes), (*n* = 3–14), ANOVA, for WT islets: *p* = 0.0054, *p* = 0.0010, *p* < 0.0001, *p* = 0.0198, *p* = 0.6772, *p* = 0.9721, *p* = 0.8916; for *Nox4* ^βKO^ islets: *P* = 0.0008, *p* = 0.0009, *p* < 0.0001, *p* = 0.9993, *p* = 0.8804, *p* = 0.7824. Only significant statistics for the relevant samples are presented. (B) cAMP quantification in WT islets (gray labeling) and *Nox4* ^βKO^ islets (red labeling) upon non‐stimulating condition (solid color), stimulation by glucose (crosshatch); glucose and glucagon (stripes); glucose, glucagon and GCGR antagonist (Cro) (filled bricks); glucose, glucagon and GLP‐1R antagonist (Ex9) (light dots); (*n* = 3–9), ANOVA, for WT islets: *p* = 0.0009, *p* < 0.0001, *p* < 0.0001, *p* = 0.0573; for *Nox4* ^βKO^ islets: *p* = 0.9997, *p* = 0.8095, *p* = 0.9998, *p* = 0.0423. Only significant statistics for the relevant samples are presented. (C) Quantification of PDE transcripts in WT (gray) and *Nox4* ^βKO^ (red) islets, (*n* = 3), statistical significance was assessed using edgeR, *p* = 0.396, *p* = 0.000149. (D) Quantification of adenylate cyclase transcripts in WT (gray) and *Nox4* ^βKO^ (red) islets, (*n* = 3), statistical significance was assessed using edgeR, *p* = 0.333, *p* = 0.0254, *p* = 0.0263. (E) Quantification of PKA subunit (regulatory, catalytic) transcripts in WT (gray) and *Nox4* ^βKO^ (red) islets, (*n* = 3), statistical significance was assessed using edgeR, *p* = 0.0571, *p* = 0.000587, *p* = 0.307, *p* = 0.591. *p ≤ 0.05, **p ≤ 0.01, ***p ≤ 0.001, ****p ≤ 0.0001.

In WT islets, glucose stimulation significantly increased intracellular cAMP production after 30 min, and this effect was further potentiated by GLP‐1 mimetic exendin‐4 and attenuated by exendin‐9‐39, but not by crotedumab (Figure 5A). In contrast, *Nox4* ^βKO^ islets exhibited elevated basal cAMP levels (*p* = 0.0040), which were further increased by exendin‐4 (Figure 5A). This response was more effectively suppressed by crotedumab than by exendin‐9‐39.

Glucagon addition under glucose stimulation enhanced cAMP production in control islets, and this effect was suppressed by GCGR inhibition but not by GLP‐1R blockade (Figure 5B). In *Nox4* ^βKO^ islets, glucagon failed to induce a cAMP increase under the same conditions. However, co‐treatment with exendin‐9‐39 significantly reduced cAMP levels (Figure 5B).

To investigate transcriptional regulators of cAMP signaling, we assessed expression of adenylyl cyclase and phosphodiesterase isoforms. Among the phosphodiesterases, the more abundantly expressed isoform *Pde3b* remained unchanged, while *Pde8b* expression was significantly increased in *Nox4* ^βKO^ islets (Figure 5C). Regarding adenylyl cyclase, *Adcy8* was upregulated and *Adcy3* downregulated in *Nox4* ^βKO^ islets, while other isoforms, such as *Adcy1*, showed no significant change (Figure 5D). Notably, *Adcy3* expression has previously been shown to be redox‐sensitive (Figure S2D). Among downstream targets, transcripts encoding PKA subunits (*Prkar1b*, *Prkar2b*) were upregulated in prediabetic *Nox4* ^βKO^ islets (Figure 5E).

These findings are consistent with altered regulation of cAMP generation and signaling components in *Nox4* ^βKO^ islets under prediabetic conditions.

## 4 Discussion

Our findings reveal that an impaired β‐cell secretory capacity triggers a compensatory response within pancreatic islets, mediated by α‐cells. Using the *Nox4* ^βKO^ prediabetic mouse model, we observed an increased α‐cell abundance, the emergence of bihormonal insulin/glucagon‐positive cells, and upregulated intra‐islet glucagon/GLP‐1 signaling components. These changes collectively support residual insulin secretion despite β‐cell dysfunction. Our data are consistent with this compensatory response involving altered engagement of glucagon and GLP‐1 receptors. Under physiological conditions, glucagon and GLP‐1 preferentially activate GLP‐1R and GCGR, respectively. In *Nox4* ^βKO^ prediabetic context, our functional data suggest a pattern of altered receptor contribution, possibly involving partial engagement of non‐preferred receptors—GLP‐1‐associated responses partially mediated via GCGR, and glucagon‐associated responses via GLP‐1R. This functional plasticity of the GLP‐1R/GCGR axis, rather than a fixed reordering of receptor hierarchy, may represent an adaptive feature of islet signaling that helps sustain β‐cell output when canonical pathways are under stress. More specifically, in the *Nox4* ^βKO^ model GLP‐1 signaling may partially engage GCGR, and glucagon signaling involves GLP‐1R. This functional flexibility of the GLP‐1R/GCGR axis may represent an adaptive mechanism to preserve β‐cell output when canonical pathways are compromised.

A key finding of our study is the selective expansion of α‐cells relative to β‐cells, accompanied by upregulation of canonical α‐cell transcription factors (*Irx1/2*, *Mafb*). While α‐cell plasticity is known in settings of severe β‐cell loss (Bramswig et al. 2013; Habener and Stanojevic 2012; Thorel et al. 2010), our data extend this to early β‐cell dysfunction, where β‐cells preserve their identity (*Pdx1*, *MafA* upregulated; *Ldha* reduced). The rise in bihormonal cells likely reflects α‐cell functional adaptation rather than β‐cell dedifferentiation. We also observed a higher proportion of large islets (> 5000μm^2^), coinciding with α‐cell expansion. This aligns with findings from Ahlgrens's 3D deep tissue imaging showing that small (~90 μm^2^), insulin‐only positive islets dominate in healthy tissue (Lehrstrand et al. 2024 ), but are selectively lost in type 1 diabetes, where larger, multihormonal islets persist (Lehrstrand et al. 2025 ). These may be more resilient in maintaining endocrine output. Supporting this, *Nox4* ^βKO^ mice maintain normoglycemia despite impaired GSIS.

We observed a disproportionate rise in glucagon production relative to α‐cell number, along with increased PC1/3 expression in a subset of α‐cells, suggesting elevated intra‐islet GLP‐1 synthesis. This aligns with clinical findings that many patients with T2D display elevated fasting glucagon levels and insufficient postprandial suppression (Grondahl et al. 2021). Despite this, meta‐analyses show that GLP‐1 levels after an oral glucose or mixed meals are not reduced in T2D, although β‐cell responsiveness to GLP‐1 is severely impaired (Calanna et al. 2013; Kjems et al. 2003). In *Nox4* ^βKO^ pancreatic tissue, we found increased levels of both GCGR and GLP‐1R, indicating higher sensitization to α‐cell signals. Recent studies have demonstrated that GLP‐1R forms nanodomains on the β‐cell membrane facing α‐cells, contributing to an earlier and robust response to stimulatory glucose, which declines during aging and metabolic stress (Tong et al. 2025). These adaptations sustain cAMP signaling even at basal glucose levels, supporting PKA and Ca^2+^ pathways essential for insulin secretion and β‐cell survival. While β‐cells primarily express GCGR in islets, both GCGR and GLP‐1R appear to be moderately expressed in δ‐cells (Svendsen et al. 2018; Adriaenssens et al. 2016), hinting at their role in intra‐islet signaling. Somatostatin from δ‐cells likely fine‐tunes glucagon/GLP‐1 pathways, with α‐cells boosting stimulation and δ‐cell modulating inhibition to sustain β‐cell function. This extends prior findings on intra‐islet GLP‐1 as an autocrine/paracrine factor under stress, revealing coordinated upregulation of ligands, receptors, and signaling—underscoring islet plasticity. Notably, the finding that mild pro‐oxidative conditions in INS‐1 cells did not recapitulate the receptor upregulation seen in vivo (Figure S2A–C) suggests that the remodeling of intra‐islet signaling in *Nox4* ^βKO^ islets reflects a systemic adaptive response to chronic β‐cell dysfunction rather than a cell‐autonomous consequence of acute redox perturbation. The redox sensitivity of Adcy3 expression (Figure S2D), however, raises the possibility that the altered cAMP signaling landscape in *Nox4* ^βKO^ islets is shaped in part by the modified redox environment, adding a layer of complexity to the interpretation of cAMP pathway changes observed in Figure 5A,B.

Although calcium imaging and receptor pharmacology experiments were conducted independently in the current study, the data are consistent with a mechanistic link between enhanced GLP‐1R/GCGR signaling and altered calcium dynamics in *Nox4* ^βKO^ β‐cells. GLP‐1R activation is well established to raise intracellular cAMP, which sensitizes L‐type voltage‐gated calcium channels via PKA‐dependent phosphorylation and potentiates K_ATP_ channel closure, collectively lowering the threshold for calcium entry and prolonging oscillatory activity (Dyachok et al. 2008). In this context, the marked transcriptional upregulation of calcium channel subunits *Cacna1a*, *Cacna1c*, *Cacna1d*, and *Cacnb2* observed in *Nox4* ^βKO^ islets (Figure 1E) may reflect a compensatory adaptation that primes β‐cells for stronger calcium responses downstream of elevated intra‐islet GLP‐1R and cAMP signaling. The persistently elevated basal cAMP levels detected in *Nox4* ^βKO^ islets (Figure 5A) are consistent with tonic GLP‐1R/GCGR activation sustaining a permissive calcium signaling environment, even as the functional coupling between calcium dynamics and insulin exocytosis remains impaired. Whether this upregulation is sufficient to restore physiological calcium oscillations, or whether distal defects in the stimulus‐secretion cascade predominate, remains to be directly tested by combined GLP‐1R modulation and calcium imaging approaches.

Human islets are characterized by a more intermingled distribution of α‐, β‐, and δ‐cells compared to rodents, which may enhance the physiological relevance of paracrine crosstalk mechanisms.

GLP‐1R and GCGR are class B G‐protein‐coupled receptors (GPCRs) with partially overlapping ligand recognition domains. Under physiological conditions, GCGR binds glucagon with higher affinity, while GLP‐1R has a higher affinity for GLP‐1 (Chepurny et al. 2019). However, diabetes is associated with receptor desensitization and altered signaling (Xu et al. 2007). Recent evidence shows that elevated glucagon levels during the development of diabetes can activate the GLP‐1R on β‐cells, promoting β‐cell regeneration and insulin secretion as part of an adaptive response (Wei et al. 2023). Our data suggest a context‐dependent shift in receptor contribution under β‐cell dysfunction. In control islets, GSIS enhancement appeared to depend predominantly on GLP‐1R. In contrast, in prediabetic islets, the GLP‐1 mimetic supported GSIS, and GCGR inhibition was associated with stronger suppression than GLP‐1R blockade across multiple conditions, consistent with altered receptor engagement. These functional differences were paralleled by changes in cAMP accumulation. Notably, glucagon‐stimulated GSIS in prediabetic islets appeared sensitive to GLP‐1R rather than GCGR blockade, suggesting a pattern of functional receptor plasticity rather than a definitive switch in signaling preference.

Interestingly, clonal β‐cells (MIN6, INS1) also show glucagon‐induced insulin secretion mediated preferentially through GLP‐1R, documenting their immature or mixed lineage characteristics. These findings are consistent with dynamic, flexible receptor engagement that may contribute to partial compensation for impaired β‐cell function. Such plasticity may reflect altered receptor density, ligand availability, receptor affinity, or biased agonism favoring signaling efficiency over classical ligand‐receptor specificity. Our findings are consistent with a revised view of glucagon as more than a counterregulatory hormone, highlighting its potential adaptive paracrine role in sustaining β‐cell output under metabolic stress.

Several limitations should be considered. First, while insulin secretion trends were biologically consistent, limited assay sensitivity under low‐insulin conditions reduced statistical power. Second, the mechanism underlying α‐cell expansion remains unresolved; lineage tracing will be required to distinguish between proliferation and transdifferentiation. Third, although we demonstrated adaptations in ligand/receptor signaling between α‐ and β‐cells, the contribution of δ‐cells to this crosstalk remains to be clarified. Fourth, while GLP‐1 and glucagon receptor signaling are conserved across species, α‐cell‐derived GLP‐1 production appears more pronounced in rodents under metabolic stress (Muller et al. 2019), potentially limiting direct translation. Nevertheless, the dispersed α‐cell distribution in human islets may enhance the relevance of such paracrine mechanisms. Moreover, our model features β‐cell‐specific *Nox4* deletion and altered redox environment. Although mild pro‐oxidative conditions did not reproduce the receptor expression changes observed in vivo (Figure S2A–C), subtle redox influences on receptor trafficking or second messenger signaling cannot be fully excluded. The redox sensitivity of *Adcy3* (Figure S2D) in particular warrants further investigation, as it may contribute to the altered cAMP dynamics observed in prediabetic islets independently of receptor‐level changes. Additionally, despite the evidence of increased paracrine alpha‐to‐beta cell signaling and upregulation of calcium channel expression, calcium dynamics, synchronization, and insulin secretion were clearly attenuated in *Nox4* ^βKO^ islets and further experiments are needed to clarify both the more proximal and distal defects in the stimulus‐secretion cascade. Finally, whether similar α‐cell‐driven compensation occurs in other models of β‐cell dysfunction or human T2D warrants further investigation.

In summary, our findings suggest that α‐cell remodeling and glucagon/GLP‐1 signaling plasticity represent candidate compensatory mechanisms that may help sustain insulin secretion during early β‐cell failure. These results highlight the therapeutic relevance of targeting intra‐islet endocrine crosstalk to preserve β‐cell function in prediabetes and early stages of T2D.

## Author Contributions

Conceptualization: L.P.‐H. Investigation and experiments: Š.B., M.K., J.D., B.H., and L.P.‐H. Data Analysis: Š.B., J.D., and L.P.‐H. Writing the original manuscript: L.P.‐H. and Š.B. Manuscript review and editing: L.P.‐H., A.S., J.D., Š.B. and B.H. Funding acquisition: L.P.‐H. L.P.‐H. is the guarantor of this work and, as such, has full access to all the data in the study and takes responsibility for the integrity of the data and the accuracy of the data analysis.

## Funding

This work was supported by the project National Institute for Research of Metabolic and Cardiovascular Diseases (Programme EXCELES, ID Project No. LX22NPO5104) to the Institute of Physiology and the Grant Agency of the Czech Republic, grant No. 26‐20384S to L.P.‐H.

## Ethics Statement

All animal experiments were ethically reviewed and performed in accordance with European Directive 86/609/EEC and approved by the Czech Central Commission for Animal Welfare.

## Conflicts of Interest

The authors declare no conflicts of interest.

## Supporting information


**Appendix S1:** cph470158‐sup‐0001‐Supinfo.zip.

## Data Availability

The datasets generated and analyzed during the current study are available from the corresponding author upon reasonable request. No applicable sources were created or analyzed during the current study.
