# Supplementary material for: Pancreatic Islet Cell Crosstalk: Insight Into α‐/β‐Cell Compensatory Mechanisms
Source: Compr Physiol. 2026 May 1;16:e70158. doi: 10.1002/cph4.70158 (PMC13135108; doi:10.1002/cph4.70158)
Supplement: Supplementary file 1 — Appendix S1: cph470158‐sup‐0001‐Supinfo.zip. [file CPH4-16-e70158-s001.zip › Benakova et al. Figures Suplement_FINAL.pdf]

List of primers used in RT-PCR gene expression quantification

**Gene name**	**forward**	**reverse**	**target**
Glp1r	GGG CCA GTA GTG TGC TAC AA	CCT CAC ACT CCG ACA GGT CC	*Mus musculus*
Gcgr	GAC AGC CCC ACC TGA ATC TC	AGG CTA GGA CAG GTT AGG GG	*Mus musculus*
Gcg	GCA CAT TCA CCA GCG ACT AC	CCT GGC CCT CCA AGT AAG AA	*Mus musculus*
Pcsk1	TCC TGT AGG CAC CTG GAC AT	TCC ACT CCT CTC CTG TCA TT	*Mus musculus*
Pcsk2	AGT CCG AAA GCT CCC CTT TG	GGT GTA GGC TGC GTC TTC TT	*Mus musculus*
Rplp0	CCC TGC ACT CTC GCT TTC TG	AAT GCC AGG ACG CGC TTG TA	*Mus musculus*
PPIA	GTG TTC TTC GAC ATC ACG GC	GCG TGT AAA GTC ACC ACC CT	*Mus musculus*
Ywhaz	ACT GAC ACT GGG CAG CAT TA	TGC GGC CCT TTT CCC AAA TA	*Mus musculus*
Hprt	CAG TCC CAG CGT CGT GAT TAG	TGA TGG CCT CCC ATC TCC TT	*Mus musculus*

Supplementary Table 1

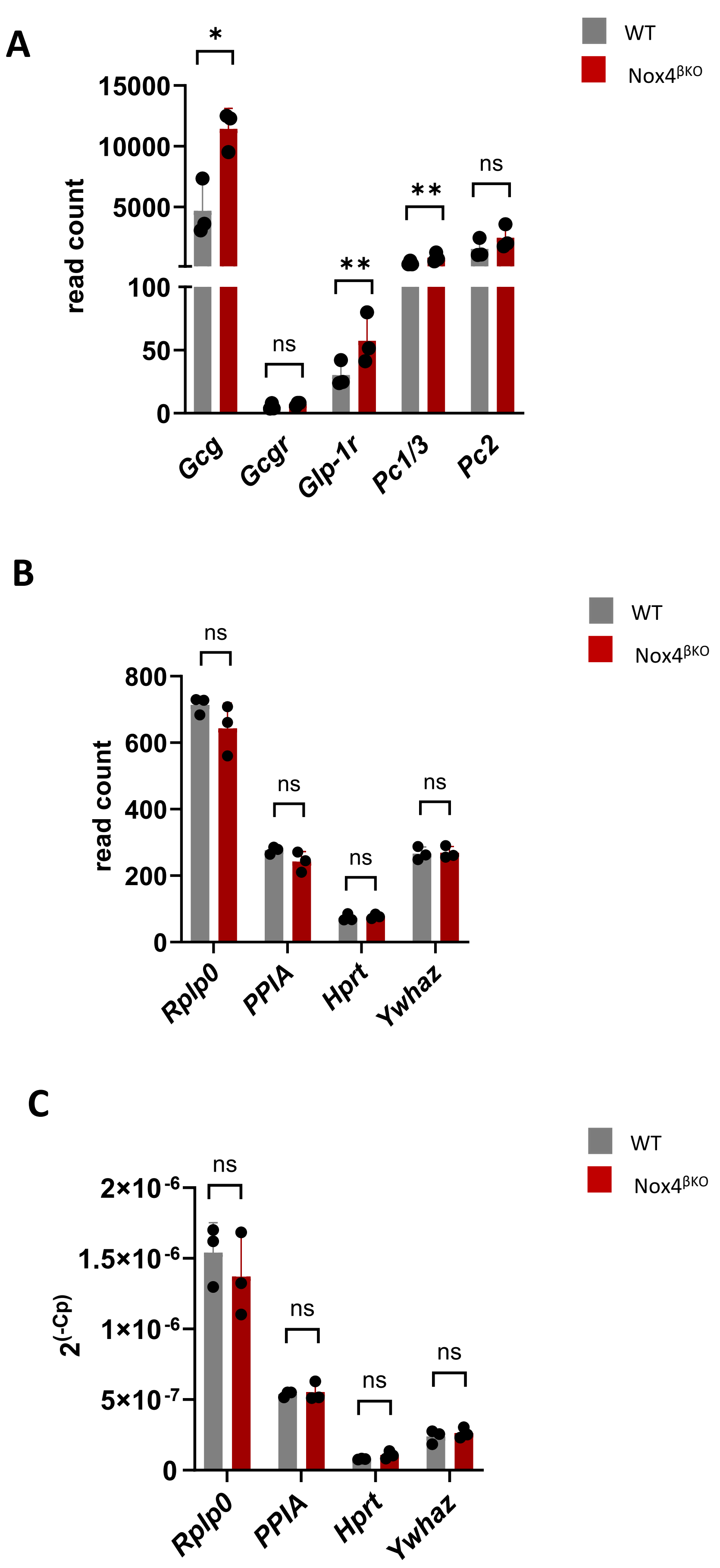

Supplementary Figure 1

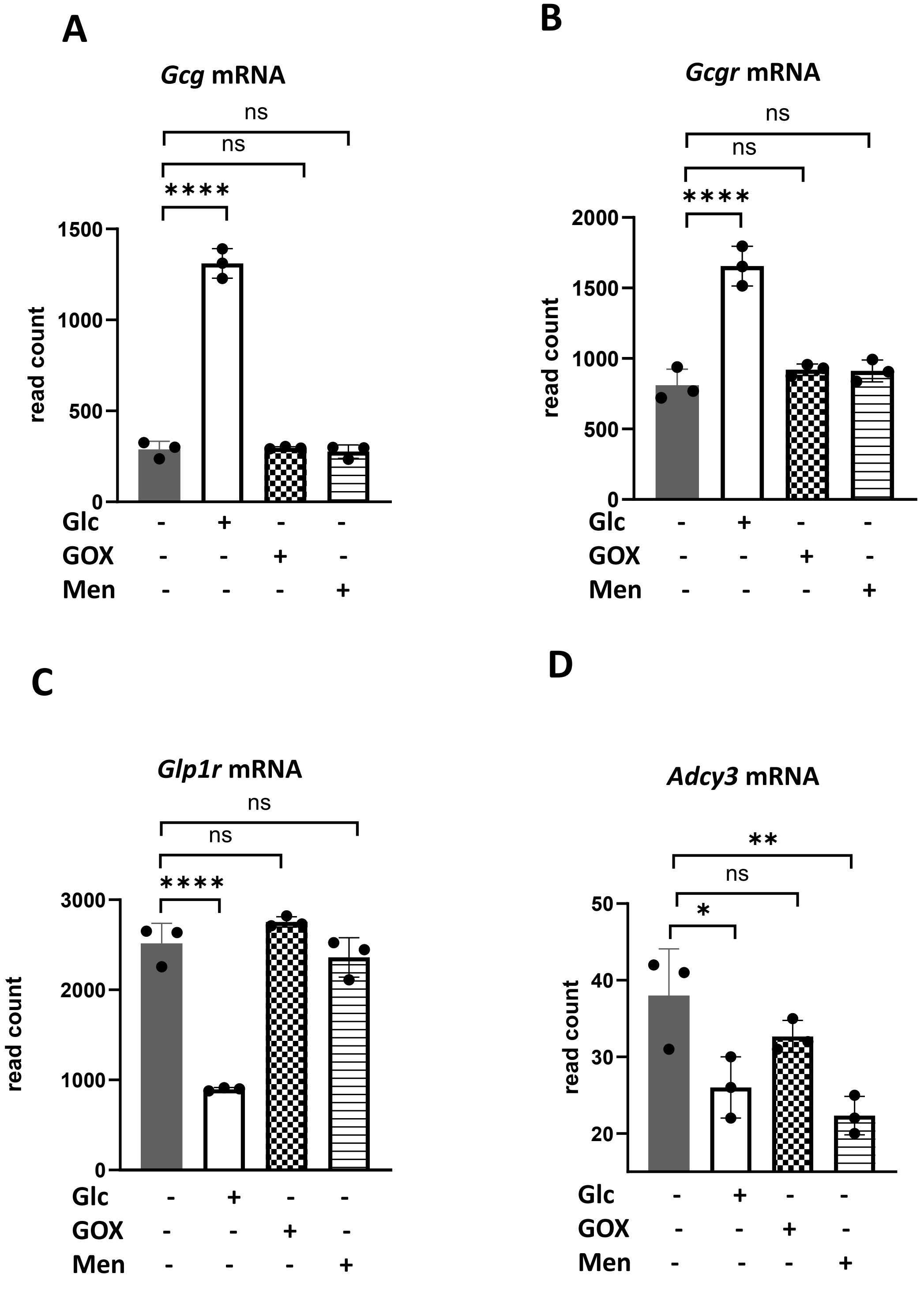

Supplementary Figure 2

A

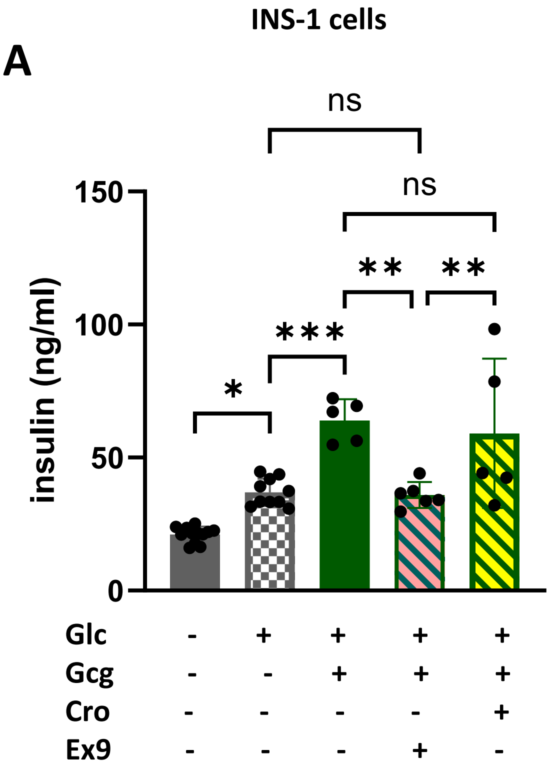

B

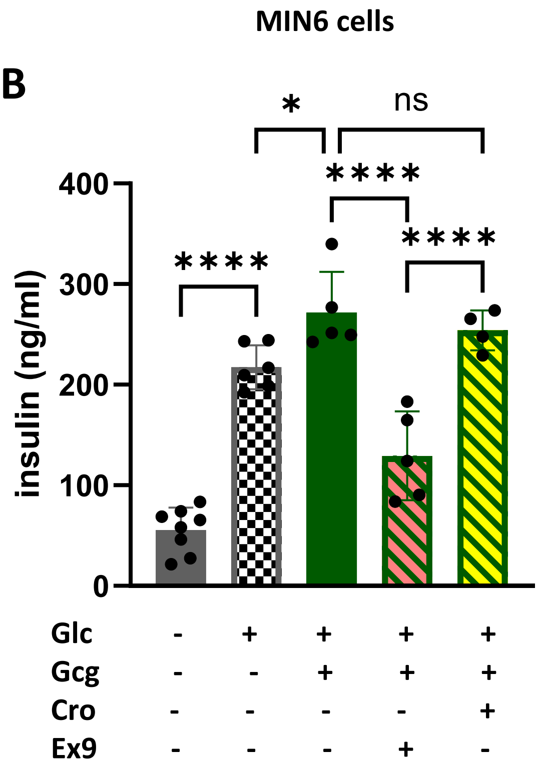

Supplementary Figure 3
