# Supplementary material for: Pancreatic Islet Cell Crosstalk: Insight Into α‐/β‐Cell Compensatory Mechanisms
Source: Compr Physiol. 2026 May 1;16:e70158. doi: 10.1002/cph4.70158 (PMC13135108; doi:10.1002/cph4.70158)
Supplement: Supplementary file 1 — Appendix S1: cph470158‐sup‐0001‐Supinfo.zip. [file CPH4-16-e70158-s001.zip › Benakova et al. Supplement FINAL.docx]

**Supplementary material**

Supplement to the manuscript entitled **Pancreatic Islet Cell Crosstalk: Insight into α-/β-Cell Compensatory Mechanisms by:** Štěpánka Benáková^1^, Blanka Holendová^1^, Jurij Dolenšek^2^, Monika Křivonosková^1^, Andraž Stožer^2^, Lydie Plecitá-Hlavatá^1^*

^1^ Department of Pancreatic Islet Research, Institute of Physiology, Czech Academy of Sciences, Prague, Czech Republic

^2^ Faculty of Medicine, University of Maribor, Maribor, Slovenia

* Corresponding author: RNDr. Lydie Plecitá-Hlavatá, PhD.

Laboratory of Pancreatic Islet Research

Institute of Physiology

Czech Academy of Sciences

Videnska 1083

142 20 Prague 4

Czech Republic

tel.: +420296442430

### **Supplementary figures legends**

**Table 1**: List of primers used in RT-PCR gene expression quantification.

**Figure S1**: **Validation of RNA-seq results by quantitative RT-PCR.** RNA-seq results were validated by quantitative reverse transcription PCR (RT-PCR). The results shown in Fig. 3 (RT-PCR) correspond to those obtained by RNA-seq. (**A**) Quantification of preproglucagon-related transcripts in wild-type (WT; grey) and Nox4βKO (red) islets (n = 3). Statistical analysis of RNA-seq data was performed using the edgeR package: p = 0.0141, p = 0.193, p = 0.00222, p = 0.00181, and p = 0.0939. (**B**) Expression of commonly used reference genes (*Rplp0*, *Ppia*, *Hprt*, and *Ywhaz*) assessed by RNA-seq (n = 3) and (**C**) by RT-PCR (n = 3), showed consistent expression patterns between methods. Statistical significance for RNA-seq was calculated by edgeR package (negative binomial model with multiple testing using Benjamini-Hochberg method): p = 0.339, p = 0.0953, p = 0.553, and p = 0.303. RT-PCR values were calculated using the 2^-Cp^ method and are presented as mean ± SD. Statistical analysis of RT-PCR results was performed using T-test: p =0.4625, p = 0.7580, p = 0.1449, p = 0.5471.

**Figure S2**: **The Effect of prooxidative environment on *Gcg, Gcgr, Glp1r* and *Adcy3* expression**. (**A**) Quantification of preproglucagon transcript in INS1 cells under non-stimulating glucose (grey), stimulating glucose (white), non-stimulating glucose and glutathione oxidase (GOX, crosshatch) and non-stimulating glucose and menadion (horizontal stripe), (n=3), ANOVA, p<0.0001, p=0.9951, p=0.9928. (**B**) Quantification of *Gcgr* transcript in INS1 cells under non-stimulating glucose (grey), stimulating glucose (white), non-stimulating glucose and glutathione oxidase (crosshatch) and non-stimulating glucose and menadion (horizontal stripe), (n=3), ANOVA, p<0.0001, p=0.5587, p=0.6173. (**C**) Quantification of *Glp-1R* transcript in INS1 cells under non-stimulating glucose (grey), stimulating glucose (white), non-stimulating glucose and glutathione oxidase (crosshatch) and non-stimulating glucose and menadion (horizontal stripe), (n=3), ANOVA, p<0.0001, p=0.3257, p=0.6519. (**D**) Quantification of *Adcy3* transcript in INS1 cells under non-stimulating glucose (grey), stimulating glucose (white), non-stimulating glucose and glutathione oxidase (crosshatch), and non-stimulating glucose and menadion (horizontal stripe), (n=3), ANOVA, p=0.0256, p=0.4121, p=0.0058.

**Figure S3**: **GSIS of glucagon-induced β-cell lines (INS1, MIN6)**. (**A**) Analysis of insulin secretion of INS1 cells upon non-stimulating condition (grey), stimulation by glucose (crosshatch); glucose and glucagon (green); glucose, glucagon and GLP-1R antagonist Exendin9-39 (Ex9) (diagonal stripe in pink bar); glucose, glucagon and GCGR antagonist crotedumab (Cro) (diagonal stripe in yellow bar); (n=6-10), ANOVA, p=0.0132p=0,0006, p=0,0013, p=0,0099, p=0,9997, p=0,9511. (**B**) Analysis of insulin secretion of MIN6 cells upon non-stimulating condition (grey), stimulation by glucose (crosshatch); glucose and glucagon (green; glucose, glucagon and GLP-1R antagonist exendin9-39 (Ex9) (diagonal stripe in pink bar); glucose, glucagon and GCGR antagonist crotedumab (Cro) (diagonal stripe in yellow bar); (n=6-10), ANOVA, p<0.0001, p=0,0489, p<0001, p<0,0001, p=0,9015.

**Methods**:

**Mild prooxidative treatment of INS1 (GOX, Menadion)** – Rat insulinoma INS-1 cells (C0018007, AddexBio, USA) were maintained under standard conditions in RPMI 1640 medium supplemented with 11 mM glucose, 10 mM HEPES, 1 mM sodium pyruvate, 5% (v/v) fetal calf serum (FCS), 50 µM 2-mercaptoethanol, 50 IU/mL penicillin, and 50 µg/mL streptomycin. For experimental treatments, the same base medium was used with glucose levels adjusted to either 3 mM or 25 mM. Cells were exposed for 24 hours to either control conditions or oxidative stress inducers - glucose oxidase (GOX, 0.1 mU/mL) or menadione (Men, 1 μM). After incubation, cells were rinsed with 1× PBS and lysed using RLT buffer (Qiagen, Germany). Total RNA was isolated following the manufacturer’s protocol using the RNeasy Mini Kit (Qiagen, Germany). Gene expression profiling was subsequently performed by high-throughput RNA sequencing (RNA-seq) to assess transcriptional changes induced by oxidative stress and varying glucose conditions.

**Glucose stimulated insulin secretion protocol in cell lines INS1 and MIN6** – INS-1 and MIN6 cells were maintained under standardized culture conditions as previously described. For GSIS experiments, cells were preincubated for 2 hours in Krebs-Ringer HEPES (KRH) buffer containing a non-stimulatory glucose concentration (3 mM) to induce glucose starvation. Following this preincubation, cells were incubated in KRH buffer containing either 3 mM or 25 mM glucose, supplemented with selected modulatory compounds targeting incretin and glucagon pathways: glucagon (50 mM), exendin 9-39 (100 nM), and crotedumab (2 nM), to modulate GLP-1R and GCGR receptor activity. After 1 hour incubation, the culture supernatants were collected and stored at –80 °C until further analysis. Insulin levels in the media were quantified using the High Sensitivity Insulin ELISA kit (62IN1PEG, Revvity, USA) following the manufacturer’s protocol.

**Calcium imaging analysis** – During off-line analysis, single β-cells were discerned using characteristic temporal activity and morphology and exported as time series using custom software (copyrights Denis Špelič). Further analysis was done using in-house MATLAB scripts. Activity of individual cells was binarized by detecting individual [Ca^2+^]_i_ oscillation in each time series. Individual oscillation was defined by time points at half maximal amplitude. Finally, binarized data was used to calculate active time (i.e., percentage of time occupied by the events, as described previously [1-3]. Furthermore, to quantify intercellular interactions and degree of synchronicity, we computed the coactivity coefficients for the binarized events, as described previously [4, 5]. In brief, for pairs of cells (i-th and j-th cell) coactivity matrix was calculated as Cij = Tij ∕√TiTj and used for assessing average coactivity across pairs of cells. Both parameters (active time and coactivity) were calculated separately for the first and the plateau phase of response for each cell. The first phase was defined by set interval during response onset (240 seconds for 8 mM and 120 seconds for 12 and 16 mM glucose) and roughly mirrored the *in vivo* peak insulin secretion (Figure 1). The plateau phase was defined as set interval (600 seconds for 8 mM, 300 seconds for 12 mM and 200 seconds for 16 mM) following stimulation onset and mirrored sustained activity.

**References:**

1. Stozer, A., et al., *Glucose-dependent activation, activity, and deactivation of beta cell networks in acute mouse pancreas tissue slices.* Am J Physiol Endocrinol Metab, 2021. **321**(2): p. E305-E323.

2. Dolensek, J., et al., *The relationship between membrane potential and calcium dynamics in glucose-stimulated beta cell syncytium in acute mouse pancreas tissue slices.* PLoS One, 2013. **8**(12): p. e82374.

3. Pohorec, V., et al., *Glucose-Stimulated Calcium Dynamics in Beta Cells From Male C57BL/6J, C57BL/6N, and NMRI Mice: A Comparison of Activation, Activity, and Deactivation Properties in Tissue Slices.* Front Endocrinol (Lausanne), 2022. **13**: p. 867663.

4. Hodson, D.J., et al., *Existence of long-lasting experience-dependent plasticity in endocrine cell networks.* Nat Commun, 2012. **3**: p. 605.

5. Dolensek, J., et al., *Ultrafast multicellular calcium imaging of calcium spikes in mouse beta cells in tissue slices.* Acta Physiol (Oxf), 2025. **241**(2): p. e14261.
